# Prediction of methicillin-resistant *Staphylococcus aureus* in patients with non-nosocomial pneumonia

**DOI:** 10.1186/1471-2334-13-370

**Published:** 2013-08-09

**Authors:** Won Jai Jung, Young Ae Kang, Moo Suk Park, Seon Cheol Park, Ah Young Leem, Eun Young Kim, Kyung Soo Chung, Young Sam Kim, Se Kyu Kim, Joon Chang, Ji Ye Jung

**Affiliations:** 1 Division of Pulmonology, Department of Internal Medicine, Severance Hospital, Yonsei University College of Medicine, 50 Yonsei-ro, Seodaemun-gu, Seoul 120-752, Republic of Korea

**Keywords:** Pneumonia, Methicillin-resistant *Staphylococcus aureus*, Non-nosocomial, Risk factors, Community-acquired pneumonia, Healthcare-associated pneumonia

## Abstract

**Background:**

Methicillin-resistant *Staphylococcus aureus* (MRSA) is recognized as an important cause of not only hospital acquired pneumonia, but also non-nosocomial pneumonia. However, the risk factors for non-nosocomial MRSA pneumonia are not clearly defined. Our objective was to identify risk factors at admission that were associated with non-nosocomial MRSA pneumonia.

**Methods:**

We evaluated 943 patients admitted to a university-affiliated hospital with culture-positive bacterial pneumonia developed outside the hospital from January 2008 to December 2011. We compared the clinical characteristics between MRSA and non-MRSA pneumonia, and identified risk factors associated with MRSA pneumonia.

**Results:**

Of 943 patients, MRSA was identified in 78 (8.2%). Higher mortality was observed in MRSA than in non-MRSA patients (33.3% *vs.* 21.5%; *P* = 0.017). In a logistic regression analysis, MRSA pneumonia was observed more frequently in patients with a previous history of MRSA infection (OR = 6.05; *P* < 0.001), a PSI score ≥120 (OR = 2.40; *P* = 0.015), intravenous antibiotic treatment within 30 days of pneumonia (OR = 2.23; *P* = 0.018). By contrast, non-MRSA pneumonia was observed more often in patients with a single infiltrate on chest radiography (OR = 0.55; *P* = 0.029).

**Conclusions:**

Anti-MRSA antibiotics could be considered in hospitalized non-nosocomial patients with several risk factors identified herein. The presence or absence of these factors would provide useful guidance in selecting initial empirical antibiotics.

## Background

Methicillin-resistant *Staphylococcus aureus* (MRSA) is the major bacterial pathogen of hospital-acquired pneumonia and ventilator-associated pneumonia, with an incidence ranging from 20% to 40% [1,2]. However, increased out-of-hospital services have led to the spread of MRSA beyond the hospital, and 2.2% to 22.3% of healthcare-associated pneumonia (HCAP) cases are caused by MRSA [3-6]. In community-acquired pneumonia (CAP), MRSA was thought to be an uncommon pathogen, comprising only 1–5% of cases [7-10]. Recently, the incidence of community-associated MRSA pneumonia has increased and it is regarded as an emerging problem [11-14].

Previous studies have reported that non-nosocomial MRSA pneumonia, including CAP and HCAP, was likely to be severe and life-threatening with a high mortality [11-15]. Therefore, recent guidelines regarding the treatment of non-nosocomial MRSA pneumonia have recommended empirical therapy for MRSA in selected patients [16-18]. However, optimal selection of such patients was not clearly described. The lack of rapid, sensitive, and specific diagnostic methods for MRSA detection in pneumonia patients makes clinicians to initiate the antibiotics treatments empirically without any degree of certainty. Some guidelines recommend that patients with particular risk factors should be started with anti-MRSA therapy; however, these recommendations are usually based on evidence from case studies or opinions of experts, resulting in inconsistencies [11-19]. Moreover, among non-nosocomial pneumonia patients, risk factors were primarily identified for CAP [20]; thus, there are limited data regarding overall characteristics and risk factors of MRSA pneumonia developed outside a hospital.

The aims of our study were to examine the proportion of pneumonia caused by MRSA among patients admitted to the hospital and to identify risk factors for MRSA at admission. This study may provide more clarified approaches for clinicians to predict MRSA pneumonia and guide initial antibiotic treatment decisions.

## Methods

### Study design and subjects

The present retrospective observational study included patients admitted with pneumonia to Severance Hospital in Seoul, South Korea between January 1, 2008 and December 31, 2011.

All patients were hospitalized with pneumonia, aged ≥ 20 years, and had culture-positive bacterial infections. Patients were classified into either non-MRSA or MRSA groups according to culture results.

The electronic medical records and imaging studies were reviewed. Data on baseline demographics, clinical manifestation, radiographic findings, and treatment outcomes were compared between MRSA and non-MRSA groups. Risk factors associated with MRSA pneumonia were identified for the prediction of MRSA pneumonia. The study protocol was approved by the Severance Institutional Review Board.

### Definitions

Pneumonia was defined as the presence of a new infiltrate on chest radiography and signs or symptoms of lower respiratory tract infection (e.g., cough, expectoration, and chest pain) that were not attributable to other causes [12]. MRSA pneumonia was defined as pneumonia coinciding with isolation of MRSA as the only potential pathogen.

HCAP was defined by at least one of the following criteria according to American Thoracic Society (ATS) and Infectious Disease Society of America (IDSA) guidelines: hospitalization within 90 days before pneumonia diagnosis; admission from a nursing home or a long-term care facility; infusion therapy such as intravenous antibiotics, chemotherapy, or wound care within 30 days before pneumonia diagnosis; and/or chronic hemodialysis or peritoneal dialysis [17].

Immunosuppressed patients were defined as those in at least one of the following categories: (1) daily use of an oral corticosteroid (≥ 15 mg prednisone/day for more than 1 month or combination therapy with low-dose steroids and other immunosuppressants, including azathioprine, mycophenolate, cyclosporine, and methotrexate); (2) radiation therapy or chemotherapy for malignancy within 6 months prior to admission; (3) infection with human immunodeficiency virus; (4) recipient of either an organ or a bone marrow transplant; or (5) underlying acquired immune deficiency disorder [20,21].

### Microbiological studies

Pathogens from respiratory specimens (bronchoalveolar lavage fluid, pleural effusion, lung abscess, or sputum) and blood were investigated using standard microbiological procedures. Sputum samples were cultured using semi-quantitative methods, and an etiological diagnosis was confirmed when a predominant microorganism was isolated from group 5 sputum, according to Murray and Washington’s grading system [22]. Positive blood cultures were considered an etiological diagnosis if no other infection source was evident. Positive urine antigen for *Streptococcus pneumoniae* or *Legionella* were considered evidence of infection. Antibodies against *Mycoplasma pneumoniae* were detected by microparticle agglutination assay (MAG). High elevated titer (> 1:160) or four-fold increase of the titer between 2–4 weeks interval is regarded as Mycoplasma infection [23]. Organisms such as coagulase-negative *staphylococci* and *diphtheroids* were considered contaminants.

### Statistical analysis

Categorical variables were analyzed using the χ^2^ test or Fisher’s exact test. Continuous variables were analyzed using *t*-test or the Mann–Whitney U test. To identify independent risk factors for MRSA pneumonia, multivariate analysis using logistic regression was conducted. Multi-collinearity of these variables was checked, and the goodness of fit of the model was verified by the Hosmer–Lemeshow test. The predictive value of the risk-scoring model was evaluated using receiver operating characteristic (ROC) curve analysis. A *P* value less than 0.05 was deemed to be statistically significant.

## Results

### Patient characteristics

Among 943 total patients, 78 (8.3%) were diagnosed with MRSA pneumonia; the remaining 865 (91.7%) comprised the non-MRSA group. Table 1 shows the baseline and clinical characteristics of the MRSA and non-MRSA groups. More patients in the MRSA group were categorized as HCAP than in the non-MRSA group (73.1% vs. 45.1%; *P* < 0.001). Diabetes, cerebral vascular accident, cardiovascular disease, and malignancy as underlying disease were more common in the MRSA group. Tube feeding, hypoxia at admission, and a previous history of MRSA infection within 1 year were more frequently observed in the MRSA group. Disease severity was more prominent in the MRSA group based on the CURB-65 and pneumonia severity index (PSI). The distribution of pathogens in non-MRSA group is presented in Table 2. Common organisms included *Pseudomonas aeruginosa* (18.5%), *Streptococcus pneumoniae* (17.7%), *Klebsiella pneumoniae* (16.5%), and *Mycoplasma pneumoniae* (15.5%).

**Table 1:** **Characteristics of patients with MRSA and non-MRSA pneumonia**^**a**^

**Characteristics**	**MRSA**	**Non-MRSA**	***P*-value**
	**(n=78)**	**(n=865)**	
Age	71.4 ± 9.3	66.0 ± 14.8	< 0.001
Male	58 (74.4)	565 (65.3)	0.106
Female	20 (25.6)	300 (34.7)	
Type of pneumonia			
Community acquired	21 (26.9)	475 (54.9)	< 0.001
Health care associated	57 (73.1)	390 (45.1)	
Underlying diseases			
Diabetes mellitus	30 (38.5)	237 (27.4)	0.038
Chronic lung disease^b^	20 (25.6)	239 (27.6)	0.706
Cerebral vascular accident	22 (28.2)	162 (18.7)	0.043
Renal disease	14 (17.9)	104 (12.0)	0.13
Hypertension	44 (9.7)	408 (47.2)	0.118
Cardiovascular disease	21 (26.9)	148 (17.1)	0.03
Liver disease	6 (7.7)	50 (5.8)	0.454
Rheumatologic disease	3 (3.8)	31 (3.6)	0.756
Malignancy	36 (46.2)	303 (35.0)	0.05
Clinical manifestation and parameters			
Bloody sputum	1 (1.3)	42 (4.9)	0.147
Confusion (decreased consciousness)	17 (21.8)	134 (15.5)	0.146
Shock at onset	20 (25.6)	267 (30.9)	0.337
Hypoxemia^c^	49 (62.8)	437 (50.5)	0.037
Acute renal failure at onset	13 (16.7)	162 (18.7)	0.654
Leukocytes /uL	12700 ± 7700	11800 ± 7300	0.341
Blood urea nitrogen > 30 mg/dL	25 (32.1)	255 (29.5)	0.634
Sodium < 130 mmol/L	15 (19.2)	132 (15.3)	0.355
pH < 7.35	7 (9)	72 (8.3)	0.843
Immunosuppressed^d^	24 (30.8)	215 (24.9)	0.25
MRSA history in previous 1 year	20 (25.6)	33 (3.8)	< 0.001
Tube feeding	12 (15.4)	64 (7.4)	0.013
CURB65	1.94 ± 1.07	1.6 ± 1.15	0.015
Pneumonia Severity Index	144.7 ± 26.1	127.6 ± 38.4	< 0.001
Admission via ER	64 (82.1)	700 (80.9)	0.808

^a^Data are presented as numbers (percentages) and plus-minus values are means ± standard deviation unless otherwise indicated.

^b^Chronic lung disease includes asthma, COPD, and structural lung diseases, such as bronchiectasis and interstitial lung disease.

^c^PaO2 < 60 mmHg, SpO2 < 90% or need for oxygen therapy.

^d^Immunosuppression includes the following: (1) daily administration of systemic corticosteroids (at least 15 mg of prednisone per day for more than one month or combination therapy with low dose corticosteroids and other immunosuppressants including azathioprine, mycophenolate, methotrexate, cyclosporine, or cyclophosphamide); (2) seropositivity for human immunodeficiency virus; (3) received either a solid organ transplant or bone marrow transplant; (4) treated with radiation therapy or chemotherapy for an underlying malignancy during the 6 months prior to hospital admission; (5) an underlying acquired immune deficiency disorder.

Abbreviations: *MRSA* methicillin-resistant *Staphylococcus aureus*, *ER* emergency room.

**Table 2:** **Distribution of isolated pathogens in patients with non-nosocomial pnseumonia**^**a**^

**Microbes**	**No. of isolates**
	**(%)**
Gram-positive pathogens	
MRSA	78 (8.3)
MSSA	51 (5.4)
*Streptococcus pneumoniae*	167 (17.7)
Gram-negative pathogens	
*Pseudomonas aeruginosa*	175 (18.5)
*Escherichia Coli*	49 (5.2)
*Klebsiella pneumoniae*	156 (16.5)
*Enterobacter* species	31 (3.3)
*Acinetobacter baumannii*	24 (2.5)
*Stenotrophomonas maltophilia*	18 (1.9)
*Haemophilus influenza*	10 (1.1)
*Moraxella catarrhalis*	24 (2.5)
Others^b^	25 (2.6)
Atypical pathogens	
*Mycoplasma pneumoniae*	146 (15.5)
*Legionella pneumophila*	2 (0.2)

^a^Data are presented as numbers (percentages). Multiple pathogens were isolated in 91 patients.

^b^*Serratia marcescens* (n=12), *Proteus mirabilis* (n=5), *Citrobacter* species (n=3), *Morganella morganii* (n=2), *Burkholderia cepacia* (n=1), *Bacteroides fragilis* (n=1), *Enterococcus faecalis* (n=1).

Abbreviations: *MRSA* methicillin-resistant *Staphylococcus aureus, MSSA* methicillin-sensitive *Staphylococcus aureus.*

### HCAP risk factors

Among the HCAP risk factors, recent hospitalization was the most common risk factor in both groups (Table 3). Patients in the MRSA group were significantly more likely to be recently hospitalized, reside in a nursing home, or receive recent intravenous antibiotics.

**Table 3:** **Comparison of HCAP risk factors between MRSA and non-MRSA pneumonia**^**a**^

**Risk factors**	**MRSA**	**Non-MRSA**	***P*-value**
Hospitalization for ≥ 2 d in the preceding 90d	50 (64.1)	313 (36.2)	< 0.001
Residence in a nursing home or extended care facility	16 (20.5)	74 (8.6)	0.001
Intravenous antibiotic treatment within 30 days	22 (28.2)	80 (9.2)	<0.001
Chemotherapy within 30 days of pneumonia	6 (7.7)	115 (13.3)	0.156
Wound care within 30 days of pneumonia	1 (1.3)	4 (0.5)	0.351
Hemodialysis clinic	2 (2.6)	30 (3.5)	1

^a^Data are presented as numbers (percentages) unless otherwise indicated.

Abbreviations: *HCAP* healthcare-associated pneumonia, *MRSA* methicillin-resistant *Staphylococcus aureus.*

### Chest radiography

On the chest radiographs, multiple infiltrates were the most common finding in the MRSA group (39.7%), and the single infiltrate was in the non-MRSA group (52%)(Table 4). The presence of a single infiltrate was significantly more frequent in non-MRSA than MRSA groups (52% *vs.* 35.9%; *P =* 0.006).

**Table 4:** **Chest radiograph findings of pneumonia patients with MRSA and non-MRSA**^**a**^

**Chest radiograph findings**	**MRSA**	**Non-MRSA**	***P*-value**
	**(n=78)**	**(n=865)**	
Single infiltrate	28 (35.9)	450 (52.0)	0.006
Multiple infiltrates	31 (39.7)	263 (30.4)	0.088
Diffuse bilateral infiltrates	19 (24.4)	152 (17.6)	0.136
Cavitation	1 (1.3)	11 (1.3)	1
Pleural effusion	16 (20.5)	126 (14.6)	0.16

^a^Data are presented as numbers (percentages) unless otherwise indicated.

Abbreviation: *MRSA* methicillin resistant *Staphylococcus aureus.*

### Treatment and clinical outcomes

From the first day of admission, glycopeptide antibiotics were administered to 14 (17.9%) patients in the MRSA group and 95 (11.0%) patients in the non-MRSA group without a significant difference (*P =* 0.065). The proportion of patients who were intubated was not different between the groups as well as the percentage of patients admitted to the intensive care unit (Table 5). However, in-hospital mortality was significantly higher in the MRSA group than in the non-MRSA group (33.3% vs. 21.5%; *P =* 0.017), and the MRSA group had a longer length of hospitalization compared with the non-MRSA group (16.5 days vs. 11 days; *P =* 0.001).

**Table 5:** **Treatment and clinical outcomes of pneumonia patients with MRSA and non-MRSA**^**a**^

**Treatment and clinical outcomes**	**MRSA**	**Non-MRSA**	***P-*value**
	**(n=78)**	**(n=865)**	
Treatment			
Intubation in ER	11 (14.1)	94 (10.9)	0.384
Intubation in overall	18 (23.1)	185 (21.4)	0.728
ICU admission via ER	11 (14.1)	152 (17.6)	0.438
ICU admission in overall	22 (28.2)	212 (24.5)	0.469
Clinical outcomes			
In-hospital mortality	26 (33.3)	186 (21.5)	0.017
Days of ICU stay, median(IQR)	15.5 (11–30)^b^	10 (3–20)^c^	0.057
Days of hospital stay, median(IQR)	16.5 (10–30)	11 (6–20)	0.001

^a^Data are presented as numbers (percentages) unless otherwise indicated.

^b^n = 22.

^c^n = 212.

Abbreviations: *MRSA* methicillin resistant *Staphylococcus aureus*, *ER* emergency room, *IQR* interquartile range, *ICU* intensive care unit.

### Factors associated with MRSA pneumonia at admission

A multivariate analysis identified three risk factors independently associated with MRSA pneumonia (Table 6). A previous history of MRSA infection within 1 year had the highest odds ratio (OR) of 6.05 (95% confidence interval [CI], 2.99–12.22; *P* < 0.001). A high PSI score (≥ 120) (OR = 2.40; 95% CI, 1.18–4.86; *P =* 0.015) and recent administration of intravenous antibiotics (OR = 2.23; 95% CI, 1.15–4.32; *P =* 0.018) were also identified as significant indicator of MRSA pneumonia. By contrast, a single infiltrate on chest radiography suggested a significantly lower risk of MRSA pneumonia (OR = 0.55; 95% CI, 0.33–0.94; *P =* 0.029).

**Table 6:** Multivariate analysis of risk factors for MRSA pneumonia

**Risk factors**	**Odds ratio**	**95% CI**	***P*-value**
Age	1.02	0.99-1.04	0.135
Hospitalization for ≥ 2 days in the preceding 90 days	1.77	0.99-3.15	0.053
Residence in a nursing home or extended care facility	1.50	0.71-3.17	0.287
Intravenous antibiotic treatment within 30 days	2.23	1.15-4.32	0.018
Tube feeding	0.85	0.39-2.15	0.853
Hypoxemia^a^	1.12	0.66-1.91	0.671
Single infiltrate	0.55	0.33-0.94	0.029
MRSA history in the previous 1 year	6.05	2.99-12.22	<0.001
PSI score ≥ 120	2.40	1.18-4.86	0.015

^a^PaO_2_ < 60 mmHg, SpO_2_ < 90%, or need for oxygen therapy.

Abbreviations: *MRSA* methicillin-resistant *Staphylococcus aureus*, *PSI* pneumonia severity index, *CI* confidence interval.

## Discussion

This is the first study performing multivariate analysis to identify risk factors for prediction of non-nosocomial MRSA pneumonia. We found that patients presenting with previous history of MRSA infection, PSI score ≥ 120, or previous intravenous antibiotics treatment within 30 days were at high risk of MRSA pneumonia and those with single infiltrate on chest radiography were at low risk.

In our study, the prevalence of MRSA as the identified etiology of non-nosocomial pneumonia was 8.3% (12.8% for HCAP and 4.2% for CAP), which was consistent with that in previous reports ranging from 3% to 30% for HCAP and 0% to 15% for CAP [24]. Because our hospital, as a large tertiary referral hospital, has many patients with prior local hospital contact, the MRSA prevalence of pneumonia is likely higher than what would be found in typical community practice. Nevertheless, our results show that MRSA is not a rare cause of CAP. The boundaries among hospital-acquired, healthcare-associated, and community-acquired MRSA pneumonia are becoming blurred because of the continuous movement of patients, and thus infections, between the hospital and community.

The current HCAP criteria include a heterogeneous group of patients with various backgrounds, particularly diverse microbial etiologies [17]. Recently, the concept of HCAP has become controversial, and several studies suggested that the definition of HCAP should be refined to improve the prediction of resistant pathogens [25,26]. In the same context, risk factors for pneumonia due to MRSA are subtly different from those for other potentially resistant pathogens and treatment for MRSA pneumonia is distinct from that for other drug-resistant pathogens. Recent IDSA guidelines recommend empirically starting antimicrobials both in cases of CAP and HCAP if certain risk factors accompany MRSA pneumonia [27]. However, the level of evidence supporting this recommendation is low with varying risk factors reported [7,11,13-15,19,27]. Therefore, a specific predicting method for MRSA pneumonia is necessary and we investigated risk factors for MRSA in all non-nosocomial pneumonia including risk factors for HCAP.

Of HCAP criteria, recent hospitalization, residence in a nursing home, chemotherapy, and dialysis were not independent risk factors for MRSA in the present study. As a tertiary university-affiliated hospital, underlying disease of malignancy was observed in more than one-third of both MRSA and non-MRSA groups, resulting in no significant difference of chemotherapy. The total number of patients on dialysis in the present study was relatively small compared with that in previous studies, so it might be an important risk factor for MRSA pneumonia in other community settings.

Besides HCAP criteria, a history of MRSA infection within 1 year and severe illness were most significant predictor of MRSA pneumonia. Known colonization or infection with MRSA is one of the most essential risk factors in MRSA pneumonia [7,27,28]. Previous studies have reported that patients with MRSA pneumonia tended to be severely ill [19,27]. Although single variables related to disease severity such as intubation, shock, and intensive care unit admission were not significantly different between the MRSA and non-MRSA groups in this study, the higher values for PSI score that represent severe disease was associated with MRSA pneumonia. Several studies reported multiple infiltrates on chest radiograph as a high risk factor for MRSA pneumonia [28], but other drug-resistant pathogens also tended to show multiple infiltrates. Thus, multiple infiltrates are not a distinct indicator for prediction of MRSA. Instead, if the patient presented with a single infiltrate on chest radiography, there was a low probability for MRSA pneumonia. Although an association between influenza and MRSA pneumonia has been established in several studies [27], we could not evaluate the relationship between them because of the low frequency of testing for influenza infection and a lack of data regarding a previous history of influenza.

Currently, there is no specific microbiological or serological diagnostic method to confirm MRSA infection immediately on presentation of non-nosocomial pneumonia. Thus, there is a need for a distinct protocol for an appropriate initial treatment for MRSA pneumonia. The identified four variables are all clinical findings available at admission and objective indices with little potential for various interpretations. Those factors could provide a clue for physicians regarding whether to start anti-MRSA treatment empirically. For years, vancomycin was the only antibiotic available for the treatment of MRSA pneumonia [1], and linezolid is an alternative choice achieving greater levels in lung epithelial lining fluid than in plasma [29]. Further study is underway to clarify which agent is more superior for the treatment of MRSA pneumonia [1].

Rapid institution of appropriate antibiotic therapy in patients with highly suspected MRSA pneumonia is strongly recommended according to several guidelines [1,18,27]. Delay of effective antibiotic therapy was associated with increased mortality in patients with ventilator associated pneumonia or septic shock [30,31]. Among the patients with MRSA bacteremia, inappropriate empirical antibiotic therapy and non-eradicable foci including pneumonia were independent risk factors for mortality [32]. However, unnecessary broad-spectrum antibiotic therapy may promote the emergence of resistant organisms in the patients and the environment. Therefore, antibiotics de-escalation should be considered according to patient’s clinical response and the final results of cultures.

Our study has several limitations. First, this was a retrospective study of patients admitted to a single center. This center is a major, university-affiliated, tertiary referral hospital in South Korea, implying that the study population was relatively severely ill and had several comorbidities. Therefore, patients in this study may not reflect those at other institutions. Physicians should consider the characteristics of their local patient population when applying our approach at their institution. Second, we analyzed only patients with culture-positive pneumonia. Culture-negative pneumonia comprised a significant proportion of all pneumonia cases. However, we included only culture-positive cases because our goal was specifically to identify MRSA risk factors. Third, a positive culture of MRSA may reflect colonization rather than true infection for some cases although we considered MRSA as the only potential pathogen. Finally, our sample size was limited to only 78 MRSA pneumonia patients; however, ours is still one of the largest sample sizes on this topic to date [7,11-15]. The prospective study with a greater number of subjects is necessary to improve prediction for MRSA pneumonia.

## Conclusion

MRSA is distinct from other drug-resistant pathogens in terms of disease severity and different antibiotic treatment. This study identified clinical risk factors that predict MRSA in hospitalized, non-nosocomial pneumonia. Anti-MRSA antibiotics could be considered in patients with several risk factors identified herein until MRSA has been excluded. The presence or absence of these factors would provide useful guidance for initial selection of empirical antibiotics.

## Abbreviations

MRSA: Methicillin-resistant *Staphylococcus aureus*; CAP: Community acquired pneumonia; HCAP: Healthcare-associated pneumonia; IDSA: Infectious Disease Society of America; ATS: American Thoracic Society; ROC: receiver operating characteristic; PSI: pneumonia severity index; CURB-65: Confusion, urea, respiration, blood pressure and age ≥65 years.

## Competing interests

The authors declare that they have no competing interests.

## Authors’ contributions

WJJ carried out screening and statistical analysis of the data and participated in the writing of the manuscript in the writing of the manuscript. SCP, AYL and KSJ carried out screening and acquisition of data. YAK, MSP, EYK and YSK participated in the study design and the analysis and interpretation of data. SKK and JC participated in the study design, analysis of data and critical revision of the manuscript for important intellectual content. JYJ participated in the study design, analysis and interpretation of data and the writing of the manuscript. All authors read and approved the final manuscript.

## Pre-publication history

The pre-publication history for this paper can be accessed here:

http://www.biomedcentral.com/1471-2334/13/370/prepub
